# Functional implications of microvascular heterogeneity for oxygen uptake and utilization

**DOI:** 10.14814/phy2.15303

**Published:** 2022-05-17

**Authors:** Tuhin K. Roy, Timothy W. Secomb

**Affiliations:** ^1^ Department of Anesthesiology Mayo Clinic Rochester Minnesota USA; ^2^ Department of Physiology University of Arizona Tucson Arizona USA

**Keywords:** flow regulation, microvascular networks, oxygen transport, vascular remodeling

## Abstract

In the vascular system, an extensive network structure provides convective and diffusive transport of oxygen to tissue. In the microcirculation, parameters describing network structure, blood flow, and oxygen transport are highly heterogeneous. This heterogeneity can strongly affect oxygen supply and organ function, including reduced oxygen uptake in the lung and decreased oxygen delivery to tissue. The causes of heterogeneity can be classified as extrinsic or intrinsic. Extrinsic heterogeneity refers to variations in oxygen demand in the systemic circulation or oxygen supply in the lungs. Intrinsic heterogeneity refers to structural heterogeneity due to stochastic growth of blood vessels and variability in flow pathways due to geometric constraints, and resulting variations in blood flow and hematocrit. Mechanisms have evolved to compensate for heterogeneity and thereby improve oxygen uptake in the lung and delivery to tissue. These mechanisms, which involve long‐term structural adaptation and short‐term flow regulation, depend on upstream responses conducted along vessel walls, and work to redistribute flow and maintain blood and tissue oxygenation. Mathematically, the variance of a functional quantity such as oxygen delivery that depends on two or more heterogeneous variables can be reduced if one of the underlying variables is controlled by an appropriate compensatory mechanism. Ineffective regulatory mechanisms can result in poor oxygen delivery even in the presence of adequate overall tissue perfusion. Restoration of endothelial function, and specifically conducted responses, should be considered when addressing tissue hypoxemia and organ failure in clinical settings.

## Graphical abstract

In the microcirculation, parameters describing network structure, blood flow, and oxygen transport are highly heterogeneous, and this heterogeneity can strongly affect oxygen supply and organ function. This review describes the compensatory flow regulation and structural adaptation mechanisms that have evolved to improve oxygen uptake in the lung and delivery to tissue. Increased understanding of the consequences of heterogeneity at the microvascular level may lead to therapeutic strategies to prevent hypoxemia and organ failure in critically ill patients.

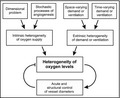

## 1 INTRODUCTION

Adequate delivery of oxygen to metabolically active tissue is the most critical demand placed on the vascular system. Lack of oxygen (hypoxia or anoxia) causes dysfunction and damage to tissue, sometimes within minutes. The mammalian cardiovascular system relies on convection to deliver oxygen via a network of blood vessels that are contained within the tissue they supply, and on diffusion from these vessels to mitochondria for ATP production. Since the typical maximum distance that oxygen can diffuse from blood vessels into oxygen‐consuming tissue is only 20–100 μm (Secomb et al., 1993a), normal oxidative metabolism and organ function require a network of tiny vessels to provide convective transport within a short distance from each cell. Tissue oxygen levels are sensitively dependent on the spatial arrangement of microvessels and the spatial and temporal distribution of flow.

**FIGURE 1:**
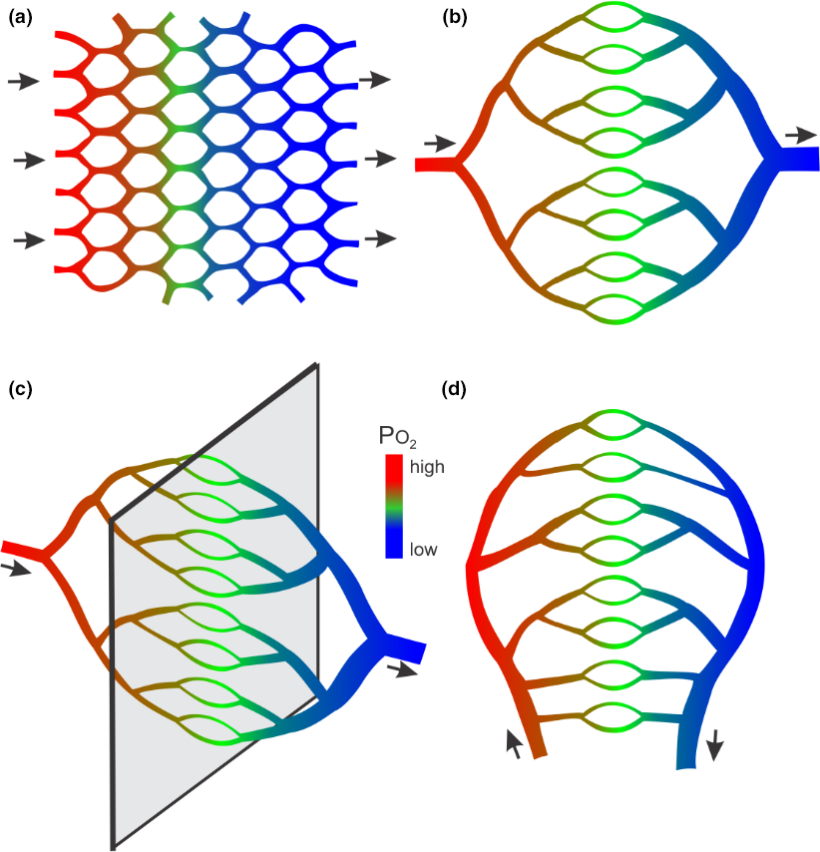
Schematic diagrams illustrating how geometric constraints and basic characteristics of mass transport processes lead to intrinsic heterogeneity in the microcirculation. Arrows indicate directions of blood flow. Color variations indicate oxygen levels in the blood. Structures shown do not represent actual microvascular network geometries. (a) The short diffusion distance of oxygen dictates that microvessels must form a fine mesh throughout the tissue. (b) High flow resistance in capillary‐sized vessels dictates that they must be fed and drained by hierarchical trees of larger‐diameter arterioles and venules, to provide efficient convective transport over larger distances. Actual microvascular networks represent a combination of these two types of structures. (c) The ‘dimensional problem’ of vascular supply. Homogeneous capillary supply to a two‐dimensional tissue region (grey sheet) can be achieved if the feeding and draining vessels form symmetric branching structures in the third dimension. If, however, the region being supplied is three‐dimensional, feeding and draining vessels must lie within the region being supplied and so such a symmetric structure is not possible. (d) Feeding and draining vessels are often adjacent, which leads to heterogeneous path lengths

According to Poiseuille's law, resistance to blood flow increases rapidly with decreasing vessel diameter. Efficient convective transport over larger distances requires larger diameter vessels, arranged in a hierarchical branching structure. Microvascular networks must therefore combine hierarchical bifurcating structures for efficient convective transport with mesh‐like structures providing short diffusion distances (Secomb et al., 2013), as illustrated in Figure 1a,b.

It was recognized many years ago that microvascular structure and flow are inherently heterogeneous, and that this has important functional consequences (Klitzman & Johnson, 1982). In 1987, Duling and Damon stated that “a clear delineation of the amount of microvascular flow heterogeneity and its control in the microcirculation is essential to our understanding of the interplay between tissue function and vascular perfusion” (Duling & Damon, 1987). This has been corroborated by theoretical simulations, which imply that tissue‐level bulk transport parameters can be significantly affected by heterogeneity (Piiper & Haab, 1991; Walley, 1996). Technical advances have facilitated the quantification of structural and functional heterogeneity (Frisbee et al., 2011a; Pries et al., 1995a, 2008; Sarelius, 1990; Zuurbier et al., 1999). The medical significance of dysfunctional distribution of blood flow and/or oxygen at the microvascular level has been increasingly recognized. Examples include “microvascular angina” (i.e., without arterial blockage, also known as cardiac syndrome X) (Cannon & Epstein, 1988; Lanza & Crea, 2010), the possible role of impaired neurovascular regulation in neurodegenerative diseases (Iadecola, 2004), and failure of local flow regulation as a cause of organ failure in sepsis (Ince, 2005). These findings have emphasized the need for better understanding of the causes of heterogeneity in the microcirculation, how heterogeneity affects exchange of oxygen, and the biological mechanisms that mitigate its effects.

**FIGURE 2:**
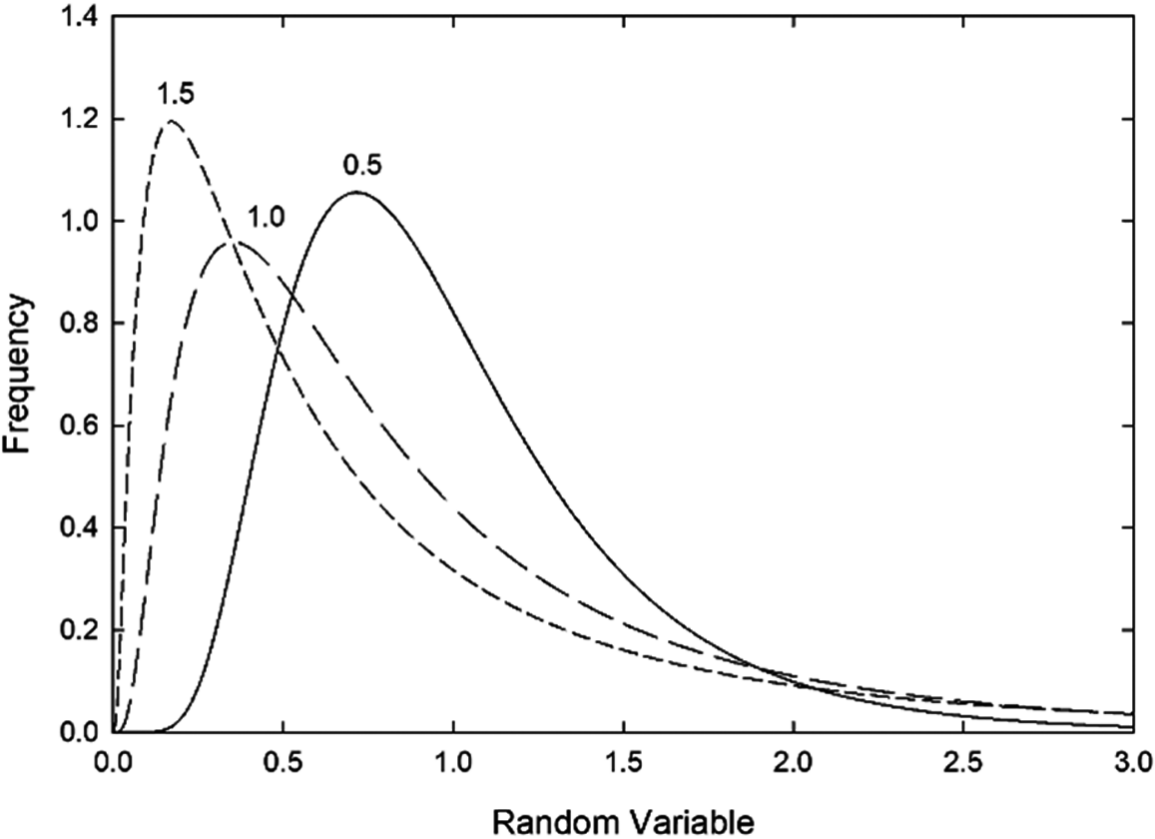
Diagram illustrating lognormal probability density functions corresponding to selected CV values as indicated, for variables with a mean of 1

For a non‐negative random variable, such as vessel diameter, length or flow rate, a simple dimensionless measure of heterogeneity is its coefficient of variation (CV), defined as the ratio of standard deviation to mean. Values of the CV derived from observations and simulations of mesenteric microvascular networks are shown in Table 1 (Pries et al., 1995b). These range up to 2 or more for flow rate and erythrocyte flux. The significance of such CV values is illustrated by Figure 2, which shows plots of lognormal distributions with CV values of 0.5, 1, and 1.5, and with mean of 1. For a CV of 1 or more, these distributions are strongly right‐skewed, with a high density of values less than the mean and a long tail of much higher values.

**TABLE 1:** Coefficients of variation (CV) for structural and functional parameters of microvascular networks. Data from (Pries et al., 1995b)

	Arterioles	Capillaries	Venules
CV of diameter	0.41	0.28	0.53
CV of length	0.83	0.65	0.82
CV of hematocrit	0.41	0.60	0.41
CV of velocity	0.84	0.99	0.65
CV of volume flow	2.23	1.76	1.86
CV of erythrocyte flow	2.22	1.92	1.86
CV of shear rate	0.92	1.62	0.97
CV of pressure gradient	1.57	2.00	1.75

**FIGURE 3:**
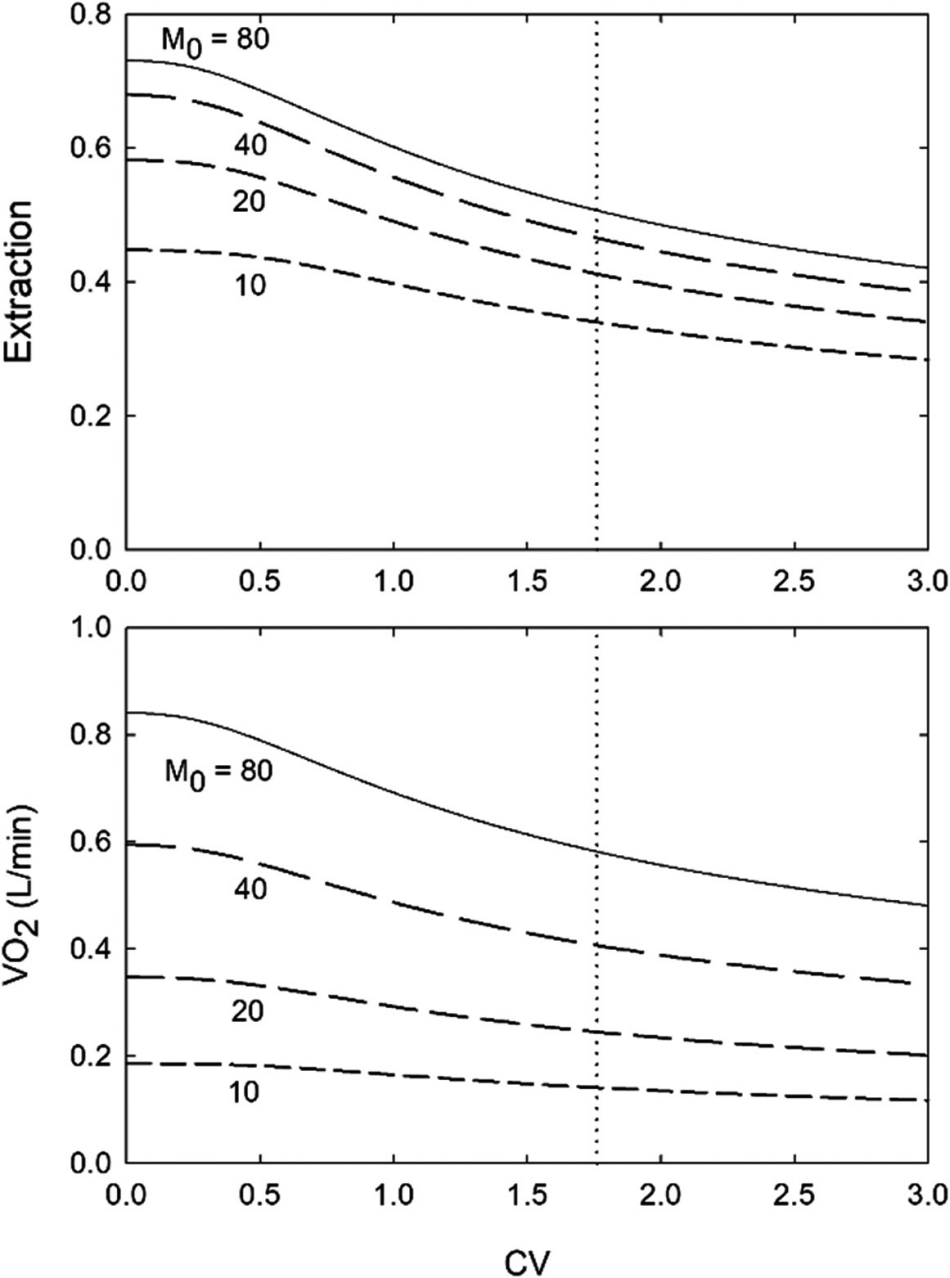
Effect of heterogeneity in blood flow, as measured by CV, on extraction and oxygen consumption. For each level of tissue oxygen demand, the CV of the flow rates is varied, while holding the total flow fixed. Simulations were conducted for a simplified representation of a tissue supplied by multiple identical parallel capillaries with heterogeneous flow rates represented by a lognormal distribution with a given CV. Oxygen transport was simulated in each cylinder using a modified Krogh cylinder model (McGuire & Secomb, 2001), taking into account the axial decline of blood oxygen content along the cylinder and assuming Michaelis‐Menten kinetics for oxygen consumption rate as a function of oxygen tension. Parameters are as specified in McGuire and Secomb (McGuire & Secomb, 2001), with the exception of a half‐maximal oxygen consumption value of 10.5 mmHg, and a capillary density of 787.25 mm^−2^ calculated as the average of values cited in the aforementioned study (McGuire & Secomb, 2001). Other parameters include a capillary radius of 2.5 µm and a nominal capillary length of 500 µm (McGuire & Secomb, 2001). Femoral muscle mass is assumed to be 2.3 kg (Andersen & Saltin, 1985). Values of muscle blood flow [L/min] corresponding to four levels of demand (80, 40, 20, and 10 cm^3^O_2_/100 cm^3^/min) were estimated based on a regression from published data (Andersen & Saltin, 1985) as 1.035 + 5.594 × $\dot{V}O_{2}$ [L/min], where $\dot{V}O_{2}$ is the calculated oxygen consumption rate. The vertical dashed line corresponds to a CV = 1.76 (see text)

To illustrate the effects of heterogeneity on transport, simulated results are presented in Figure 3 for oxygen delivery by an array of Krogh cylinders with a lognormal distribution of flows, characterized by CV. Details of the simulation are provided in the caption. As the CV is increased, oxygen consumption and extraction decrease, because tissue becomes hypoxic in regions with low blood flow rates. Observations in mesentery indicate CV = 1.76 for capillary flow (Pries et al., 1995b). The corresponding reduction in oxygen consumption, relative to CV = 0, ranges from 24% at low oxygen demand to 31% at high oxygen demand. This simulation suggests that heterogeneity in the microcirculation cannot be regarded simply as random “noise” superimposed on functional variables, but is itself an important determinant of oxygen transport properties. In the lung, simulating heterogeneity of flow at the alveolar level similarly decreases the predicted effective diffusing capacity for oxygen (Roy & Secomb, 2019).

**FIGURE 4:**
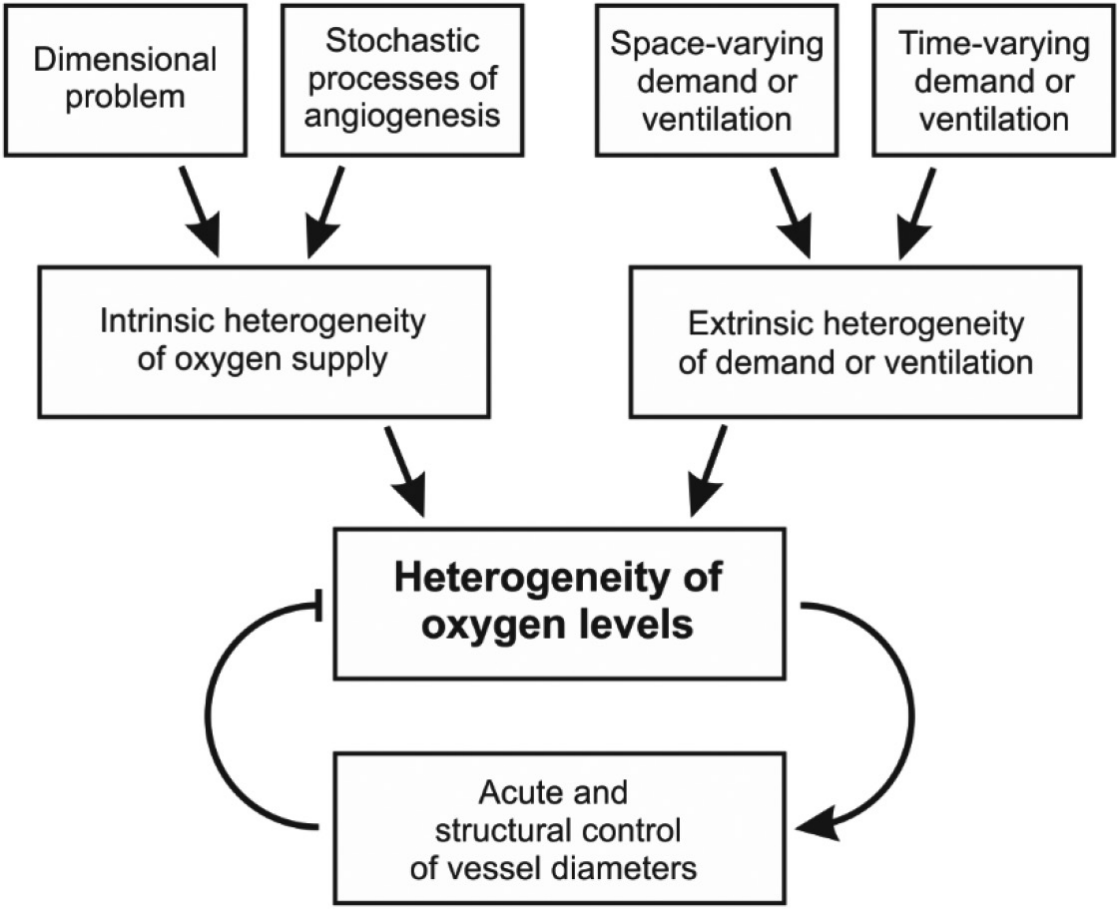
Schematic representation of factors contributing to the heterogeneity of tissue oxygen levels. Arrow with blunt end represents negative effect. Microvascular networks have heterogeneous structures as a result of the “dimensional problem” and the stochastic processes of angiogenesis, with the consequence that oxygen supply is intrinsically heterogeneous. Oxidative metabolism in tissues and ventilation in the lung vary in both space and time, causing extrinsic heterogeneity in tissue oxygen levels. Multiple control mechanisms act on both short and long time scales to mitigate this heterogeneity and ensure adequate tissue oxygenation under normal conditions

In this review, we discuss the origins of heterogeneity, its effects on oxygen transport, and the mechanisms by which its effects are mitigated (Figure 4). The perspective of Pries & Secomb (2009) is adopted, according to which heterogeneity of tissue oxygen levels is an inevitable consequence of the physiological functions of the vasculature, the mechanisms of vascular growth, and geometrical constraints. In a normally functioning organ, active mechanisms of flow regulation and structural adaptation allow adequate organ function by compensating for this heterogeneity, while also adjusting local flows in response to spatial and temporal variations in metabolic demand. In the lung, the local matching of perfusion to varying levels of ventilation is required to ensure adequate oxygen uptake and maintain arterial oxygen saturation. In pathophysiological states or aging, decreased ability to compensate for heterogeneity contributes to functional deficits and eventual organ dysfunction. Better understanding of these phenomena may therefore provide a basis for approaches to improve oxygen delivery and organ function in such conditions.

## 2 ORIGINS OF MICROVASCULAR HETEROGENEITY

Heterogeneity at the microvascular level can be broadly classified as *extrinsic*, arising in response to variations in metabolic demand in the systemic circulation or ventilation in the lung, or *intrinsic*, resulting from the inherent characteristics of microvascular structures.

### 2.1 Extrinsic heterogeneity

Most tissues of the body are subject to transient increases in oxygen demand above baseline to meet functional requirements. Skeletal muscle, heart, brain, kidney, liver, and intestinal mucosa all exhibit periods of increased activity. The resulting transient increases in oxygen demand may be spatially heterogeneous, as for example when a subpopulation of motor units is activated in muscle. The term *extrinsic heterogeneity* is used here to refer to spatial and temporal variations in functional characteristics of the tissue, such as oxygen demand in systemic tissues and oxygen availability in the lung.

In skeletal muscle, the differential characteristics of muscle fiber types in terms of recruitment and oxygen demand at different levels of exercise (e.g. increased recruitment of fast twitch fibers at higher running speeds) can cause both spatial and temporal heterogeneity as exercise is initiated (Koga et al., 2014); this is corroborated by measurements demonstrating spatial heterogeneity in quadriceps deoxygenation with exercise (Koga et al., 2011).

Increases in systemic oxygen consumption (such as during exercise) result in an increased heart rate and contractility, with a resultant increase in oxygen demand in the heart. The increase in heart rate results in a higher proportion of time in systole, and subendocardial vessel compression results in decreased blood flow during muscle contraction (Duncker et al., 2015), creating a spatial gradient from endocardium to epicardium in systolic perfusion.

In the brain, oxygen demand varies according to neural activity; these variations can occur at the regional or local level and have been documented using functional MRI (Buxton, 2010) and confocal microscopy (Yaseen et al., 2011). This process, termed neurovascular coupling, augments oxygen delivery to accommodate demand (Girouard & Iadecola, 2006; Weber et al., 2015). Extrinsic heterogeneity in flow can also result from occlusions of the microvasculature due to microthrombi (Hu & Stiefel, 2016; Molina, 2011).

The kidney, liver, and gut all exhibit a high degree of variability in oxygen consumption due to temporal changes in demand (e.g., toxin clearance or postprandial transport). The kidney is unique in its compartmentalization, with its cortex functioning to filter and absorb solutes and the medulla responsible for water resorption. The bulk of blood flow goes to the cortex, which maintains much higher oxygen tensions (Molema & Aird, 2012). Renal oxygen consumption is dependent in part on the rate of sodium resorption, which requires ATP (Lee, Gardiner, et al., 2017; Lee, Ngo, et al., 2017). The kidney is susceptible to hypoxia, which is associated with acute and chronic kidney disease (Ow et al., 2018).

As an organ of oxygen uptake, the lung exhibits considerable heterogeneity in terms of oxygen availability in different regions. Both ventilation and perfusion are heterogeneous on different scales, with ventilation/perfusion ratios differing in dependent portions of the lung (West, 1969, 2004). The large‐scale effect of gravity is exploited in critically ill patients by using prone positioning to restore ventilation to atelectatic portions of the lung in conditions such as ARDS (Johnson et al., 2017; Lamm et al., 1994). The structure of the lung, with its lobes and secondary lobules, introduces spatial heterogeneity in oxygen supply, since vessels from the bronchial circulation follow this organization. At smaller scales, the branching structure leads to decreased flow in the distal part of the tracheobronchial tree and can lead to diffusional screening of oxygen according to theoretical predictions (Felici et al., 2003, 2005; Sapoval et al., 2002).

### 2.2 Intrinsic heterogeneity

The term *intrinsic heterogeneity* is used here to refer to variations in oxygen supply as a consequence of the inherent characteristics of vascular network structure, including segment lengths, diameters, mechanical properties such as stiffness, and connectivity, as well as the particulate nature of blood.

The locations and dimensions of larger vessels in mammalian circulatory systems are largely genetically predetermined. In contrast, the microcirculation is a largely self‐organizing structure, in which the processes of angiogenesis, structural remodeling, and pruning of redundant vessels are governed by a set of generic responses to local stimuli (Pries & Secomb, 2014; Secomb et al., 2013), resulting in heterogeneous structures (Frisbee et al., 2011b; Pries et al., 1995b, 2008; Sarelius, 1990; Zuurbier et al., 1999).

One inherent source of structural heterogeneity derives from the embedding of a branching network within the same region that is supplied. This “dimensional problem” (Pries & Secomb, 2009) can be appreciated by considering that a two‐dimensional region can be readily supplied by a three‐dimensional symmetrically branching vascular network, in which all pathways and flows are equivalent (Figure 1c). If, however, the region to be supplied is three‐dimensional, this cannot be achieved, and non‐equivalent flow pathways must be present. Furthermore, feeding and draining vessels are often adjacent to each other, creating more heterogeneity among flow pathways (Figure 1d).

Because of the dependence of flow resistance in blood vessels on length and diameter in accordance with Poiseuille's law (Pries et al., 2008), wide variations in flow occur due to the structural heterogeneity of microvascular networks (Duling, 1994; Duling & Damon, 1987; Ellsworth et al., 1988; Klitzman & Johnson, 1982; Pries et al., 1996). Heterogeneity in erythrocyte fluxes can be exacerbated by the particulate nature of blood (Secomb, 1991), as manifested by the phenomenon of phase separation. This uneven partitioning of hematocrit at diverging bifurcations occurs because erythrocytes preferentially enter the branch with higher flow. Consequences include wide variations in microvessel hematocrit, viscosity, and flow (Pries et al., 1986, 1990, 2008) and increased heterogeneity of oxygen delivery. If a daughter branch has zero hematocrit, a plasma channel is formed. The likelihood of plasma channel formation increases with anemia or hemodilution (Pries et al., 1992), leaving surrounding tissue hypoxic (Morisaki et al., 1996). Vasomotion (Aalkjaer et al., 2011; Arciero & Secomb, 2012), i.e., spontaneous oscillations in vessel diameter, can result in temporal variations in blood flow and oxygen transport.

Microvascular networks in solid tumors are highly heterogeneous in their structure, blood flow, and oxygenation characteristics. This abnormal heterogeneity may result from impaired structural adaptation mechanisms, and in particular from lack of normal conducted responses in tumor vasculature due to dysfunctional communication between endothelial cells (Pries et al., 2009). Phase separation at successive bifurcations leads to significant numbers of vessels with few or no erythrocytes (Dewhirst et al., 1996). In normal healthy tissues, regulatory mechanisms act to reduce the occurrence of plasma channels, but tumor microvessels with dysfunctional regulation demonstrate high numbers of such vessels. Altered patterns of VEGF secretion in tumors stimulate abnormal patterns of angiogenesis, leading to regions of low flow and hypoxia even under conditions of high overall perfusion (Gillies et al., 1999). Heterogeneous flow distributions, combined with increases in vascular permeability and impairment of conducted responses responsible for effective flow regulation, results in regions of hypoxia and acidosis that can limit the effectiveness of radiation and chemotherapeutic agents (Dewhirst & Secomb, 2017). Simulations suggest that temporal fluctuations in oxygenation can also have implications for therapy, since transient hypoxia may coincide with treatment such as chemotherapy and radiation (Cardenas‐Navia et al., 2007).

A number of disease states including hypertension (Feihl et al., 2006; Triantafyllou et al., 2015), the metabolic syndrome (Paavonsalo et al., 2020), and renal disease (Afsar et al., 2018) are associated with capillary rarefaction, a reduction in capillary density. The resulting decrease in blood flow due to increased vascular resistance can impair oxygen delivery and make the tissue more vulnerable to hypoxia as a consequence of microvascular heterogeneity (Tickle et al., 2020).

More generally, heterogeneity in vascular reactivity may be due in part to variations in the receptors and channels involved in flow regulation, such as potassium and calcium channels expressed in different vascular beds and in pathophysiological states, as well as differences in adrenoceptor distribution (Davis et al., 2008). Passive mechanical properties of microvessels may also be heterogeneous and this would contribute to heterogeneity in their vasoactive responses.

### 2.3 Quantification of heterogeneity in flow and oxygen delivery

Heterogeneity can be quantified by examining the distributions of variables related to structure, hemodynamics, and oxygen levels. For structural heterogeneity, relevant variables are segment lengths, segment diameters, and path lengths. For hemodynamic heterogeneity, relevant variables include segment flow rates and hematocrits (Pries et al., 1995b, 1996). As discussed in the Introduction, the coefficient of variation (CV) is a basic measure of heterogeneity in these variables. Measures of heterogeneity may vary according to the spatial resolution at which measurements are made. Studies of regional myocardial blood flow (Bassingthwaighte et al., 1989) showed that the variability of flow increased when assessed at progressively smaller scales, and could be characterized using a fractal dimension.

The distribution of oxygen tensions within tissue is a critical determinant of organ function. A common measure of tissue hypoxia is the hypoxic fraction, i.e., the fraction of tissue at an oxygen tension under a critical value, below which tissue function is significantly limited by lack of oxygen. Experimental determination of tissue oxygen levels is challenging because steep gradients exist on microscopic scales. Theoretical simulations of oxygen transport can be used to predict the spatial distribution of oxygen levels on microvascular scales (Secomb et al., 1993b, 2000, 2004).

The multiplicity of flow pathways results in a distribution of transit times for blood passing through a tissue. The standard deviation of transit times, termed capillary transit time heterogeneity (CTH), has been proposed as a means to quantify the effects of heterogeneity on tissue oxygenation (Jespersen & Ostergaard, 2012). Simulations suggest that CTH increases in pathophysiological conditions (Jespersen & Ostergaard, 2012), resulting in functional deficits in the brain (Angleys et al., 2015; Ostergaard et al., 2014). The use of CTH as a functional measure of heterogeneity is supported by consideration of the effects of transit time on oxygen transport. Long transit times result in depletion of intracapillary oxygen and hypoxia, whereas short transit times result in a low level of oxygen extraction. However, reduction of CTH does not necessarily imply improved tissue oxygenation. In a heterogeneous network, optimal oxygen delivery would require variation in capillary transit times to compensate for heterogeneity of capillary diameters and inter‐capillary distances.

An alternative measure of oxygen supply variation that has been used in theoretical modeling is the capillary outflow saturation heterogeneity, or COSH (Lucker et al., 2018a, 2018b). This is proposed as a more direct measure of heterogeneity in tissue oxygenation as compared to CTH. Capillary outflow saturation is representative of tissue oxygen levels, since capillary oxygen tensions are approximately equilibrated with tissue values. A COSH value near zero implies close matching between perfusion and metabolic need on all flow pathways, and minimizes tissue hypoxia for a given level of total perfusion.

### 2.4 Mathematical theory for heterogeneity of combined variables

Heterogeneity in functional parameters of interest, such as capillary flow rates, results from variations in underlying variables including vessel diameters, lengths, and hematocrits. The resulting functional heterogeneity depends not only on the variances of the underlying variables, but also on their covariances (Vicaut, 1986). The effects of covariances on the variance of combined variables can be illustrated by considering two random variables X and Y. The variances of their sum and product are given by:

$$\text{var}\left(X+Y\right)=\text{var}\left(X\right)+\text{var}\left(Y\right)+2\;\text{cov}\left(X,Y\right)$$

$$\begin{array}{cc} \text{var}\left(XY\right)\; & ={\bar{X}}^{2}\text{var}\left(Y\right)+{\bar{Y}}^{2}\text{var}\left(X\right)+\text{var}\left(X\right)\text{var}\left(Y\right)+\text{cov}\left(X^{2},Y^{2}\right)\\ & \;‐{\left[\text{cov}\left(X,Y\right)\right]}^{2}‐2\bar{X}\;\bar{Y}\;\text{cov}\left(X,Y\right) \end{array}$$

where the overbars represent the expected value. In general, the term $\text{cov}\left(X^{2},Y^{2}\right)$ dominates the last two terms in the formula for the product. Analogous relationships apply for multiple‐term products (Goodman, 1962).

If X and Y are uncorrelated, then the variances of X+Y and XY include contributions from the variances of X and Y. The overall variance increases due to the combined variances. If X and Y are positively correlated, then the variance of the combined variables is further increased. For example, vessel hematocrit typically shows a positive correlation with flow rate in a population of vessels due to the effects of phase separation at diverging vessel bifurcations. The erythrocyte flux is proportional to the product of hematocrit and flow rate, and this mechanism acts to increase its variance.

As discussed below, multiple mechanisms for local control of blood flow mitigate the adverse effects of heterogeneity on tissue oxygenation. Suppose that a random variable X represents the underlying heterogeneity, and Y represents a controlled variable, such as vessel flow conductance, that acts to mitigate the effects of variations in X. In this case, cov(X,Y) and $\text{cov}\left(X^{2},Y^{2}\right)$ are typically negative and decrease the variance of the resulting functional variables X+Y and XY, according to the above formulae. This provides a mathematical basis for the ability of control mechanisms to mitigate heterogeneity.

## 3 CONTROL MECHANISMS AND THEIR ROLE IN MITIGATING HETEROGENEITY

Mechanisms for local control of blood flow mitigate the intrinsic heterogeneity of the microcirculation and accommodate temporal and spatial changes in demand or ventilation. In the following sections, contrasting characteristics of various flow control mechanisms are described, with reference to their effects on heterogeneity.

### 3.1 Acute versus chronic mechanisms

Acute control of blood flow is achieved primarily by changes in diameters of arterioles and small arteries, effected by modulating smooth muscle tone (Davis et al., 2008). At the capillary level, diameter change by contractility of endothelial cells or pericytes may also play a role (Gonzales et al., 2020; Hamilton et al., 2010; MacDonald et al., 1995; Ragan et al., 1988). Resulting changes in blood flow occur over seconds to minutes. Longer term alterations in blood flow over hours to days occur via structural remodeling, including angiogenesis and pruning as well as changes in arteriolar and venular vessel diameter and wall mass (Secomb et al., 2013). Persistent arteriolar vasoconstriction or dilation can lead to corresponding structural remodeling of vessel diameters (Bakker et al., 2000, 2008; Martinez‐Lemus et al., 2004, 2009). Because of this linkage between vasoactive and structural responses, acute and chronic control mechanisms can act in parallel to control tissue perfusion and mitigate heterogeneity in tissue oxygenation.

### 3.2 Metabolic versus hemodynamic mechanisms

A control system can be viewed as a combination of sensors that detect physiological stimuli and effectors that can modulate flow. In microcirculation, the control system must match flow to metabolic needs while maintaining the hemodynamic efficiency of the system. Therefore, both metabolic and hemodynamic stimuli must be sensed. The resulting signals are integrated in the vessel walls and drive responses via effector mechanisms, which include changes in vascular smooth muscle tone and restructuring of vessel wall components.

Ensuring adequate oxygen supply to all parts of tissue, despite the heterogeneity of both supply and demand, requires local sensitivity to metabolic conditions. Hypoxia results in acute vasodilation in many tissues, and over time leads to vascular remodeling and/or new vessel growth via angiogenesis. Acute local control of arteriolar diameters occurs in part via oxygen‐dependent vasoconstriction or dilation. Multiple tissues and blood oxygen‐dependent mechanisms with various mediators have been implicated in different tissues, although details are not well understood (Jackson, 2016; Liu et al., 2017). Long‐term control of vessel diameters also occurs via oxygen dependence, with growth factors including VEGF produced in response to hypoxia (Reglin & Pries, 2014).

Appropriate hemodynamic conditions must be maintained in the microcirculation to ensure that metabolic needs are met efficiently. According to Murray's law, the flow should be proportional to the cube of diameter in order to minimize the “cost” of pumping, which consists of the rate of mechanical work and a term proportional to blood volume (LaBarbera, 1990; Murray, 1926a, 1926b; Sherman, 1981). This condition is satisfied if wall shear stress generated by blood flow is approximately constant throughout the network. Wall shear stress is sensed by endothelial cells and contributes to both short‐term and long‐term control of vessel diameter. Increased shear stress results in acute vasodilation (Pohl et al., 2000) and chronic outward remodeling (Kamiya et al., 1984; Rodbard, 1975).

Analogously, acutely increased intravascular pressure results in vasoconstriction by the myogenic response (Johnson et al., 1991), while chronic increases in pressure lead to inward remodeling of vessels (Heagerty et al., 1993). The law of Laplace implies that the dominant stress in vessel walls resulting from intravascular pressure is circumferential stress. Changes in pressure are sensed via circumferential stress in vessel wall components. Mathematical models suggest that the myogenic response to pressure is an important contributor to the autoregulation of blood flow (Carlson et al., 2008). Inward remodeling in response to high pressure ensures that flow resistance is higher on the arterial side than the venous side of the systemic circulation. This has the important consequence of lowering capillary pressures and limiting fluid exchange between capillaries and tissue according to the Starling equation (Pries et al., 2001).

**FIGURE 5:**
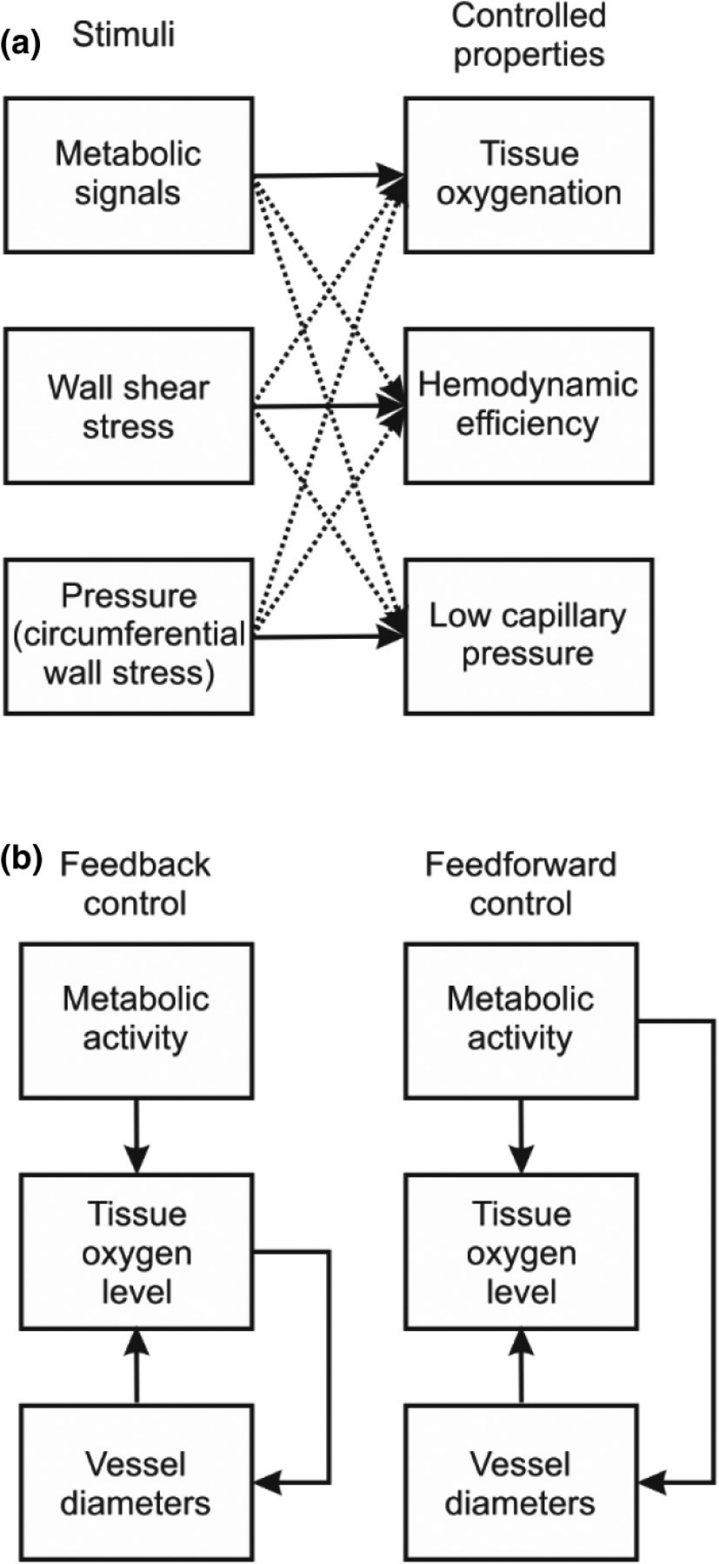
Schematic representation of control mechanisms in the circulatory system. (a) Interaction of metabolic and hemodynamic control mechanisms. The solid arrows indicate the property that is primarily controlled by responses to each type of stimulus. All control mechanisms act via changes in vessel diameters, and therefore influence all of the controlled properties. This cross‐talk is represented by the dashed lines. Because of this cross‐talk, multiple variables cannot be controlled individually by a single mechanism (variation in vessel diameters), and some heterogeneity in controlled properties is inevitable. (b) Illustration of feedback and feedforward mechanisms for control of tissue oxygenation. In feedback control, changes in oxygen levels drive changes in vessel diameters. In feedforward control, signals derived directly from the metabolic activity drive changes in vessel diameters

These regulatory mechanisms are inherently limited in their ability to achieve homogeneous tissue oxygen levels. Any control mechanism based on sensing oxygen levels requires a finite signal to generate responses, since it cannot have infinite gain. Therefore, precise control of oxygen levels to a preset level is not possible. Moreover, vessels are responding to a combination of hemodynamic and metabolic signals. The metabolic signals are heterogeneous, which implies heterogeneity in the responses of vessels to a given oxygen‐dependent signal. The oxygen content of arterioles and capillaries generally declines in the downstream direction, as oxygen is delivered. Tissue oxygen levels decline with radial distance from vessels, providing a gradient for diffusion. The relationship between hemodynamic and metabolic control mechanisms is illustrated schematically in Figure 5a.

### 3.3 Feedforward versus feedback mechanisms

The classical paradigm of homeostasis is prevalent in physiological reasoning. According to this principle, departures of state variables from desired levels result in stimuli that tend to restore the variable to its desired level, implying the existence of feedback control mechanisms. The metabolic and hemodynamic responses, as discussed above, are examples of feedback control for oxygen levels and wall shear stresses.

Feedforward mechanisms represent a distinct and important mode of control in the circulatory system. In feedforward control, a signal other than hypoxia associated with increased metabolic demand provides the stimulus for an increase in blood flow. Typically, this signal appears in advance of the decrease in oxygen levels resulting from the increased metabolic activity. A feedforward mechanism generally allows a faster response than a feedback mechanism, and can prevent or even reverse an unfavorable change in the controlled variable, such as a decrease in tissue oxygen levels. Control by feedback and feedforward mechanisms is illustrated schematically in Figure 5b.

In several tissues, increases in metabolic demand are triggered by neural activity, and the associated rapid release of potassium ions and neurotransmitters into the extracellular space provides potential signals for feedforward control. In skeletal muscle, the sensing of increased interstitial potassium upon stimulation has been shown to contribute to the vasodilator response (Armstrong et al., 2007). Simulations indicate that sensing of potassium concentration by capillaries may increase blood flow via upstream conducted signals in anticipation of increased oxygen demand (Lo et al., 2004).

In the cerebral cortex, the increase in blood flow associated with increased brain activity is termed neurovascular coupling. The relative increase in flow is observed to be larger than the relative increase in oxygen consumption (Masamoto et al., 2008). In spite of the fact that the brain is highly intolerant of hypoxia, neurovascular coupling is not dependent on changes in oxygen levels (Leithner & Royl, 2014). Experimental evidence shows arterial and arteriolar dilation in response to somatosensory stimulation (Drew et al., 2011). These observations imply that feedforward control plays a major role in neurovascular coupling.

In the heart, sympathetic activation of α‐adrenoreceptor‐mediated vasoconstriction as well as β‐adrenoreceptor mediated vasodilation result in a net increase in supply in a feedforward manner independent of oxygen levels in anticipation of increased metabolic demand in the heart (Tune et al., 2020). Coronary sinus oxygen tension has been shown to improve with alpha‐blockade but decrease with combined alpha‐ and beta‐blockade relative to control conditions, suggesting that feedforward vasodilation plays an important role in cardiac metabolism‐perfusion matching (Gorman et al., 2000).

The characteristics of feedforward control make it well suited to provide a rapid response to time‐varying metabolic demands driven by extrinsic factors. However, it does not respond directly to the controlled variable, tissue oxygenation. Therefore, feedforward control must act in concert with oxygen‐sensitive feedback mechanisms to prevent tissue hypoxia, given that oxygen supply and demand are heterogeneous. Since brain tissue is intolerant of hypoxia, the lack of acute vascular responses to hypoxia in brain is surprising (Leithner & Royl, 2014). Simulations suggest that a possible resolution to this apparent paradox may be that the long‐term processes of angiogenesis and structural adaptation are sensitive to oxygen levels (Alberding & Secomb, 2021), ensuring that tissue is well oxygenated under resting conditions.

### 3.4 Positive versus negative feedback

Although homeostasis in physiology is generally considered to be achieved by means of negative feedback mechanisms, normal biological responses can also result in positive feedback loops in hemodynamic control.

Small arteries and arterioles generally respond to increased internal pressure by inward remodeling. This response plays an important role in homeostasis by controlling capillary pressure, as already discussed. However, this mechanism introduces positive feedback in the response to an increase in arterial pressure. The resulting inward remodeling increases peripheral resistance and requires still higher arterial pressures to achieve adequate perfusion. This mechanism is hypothesized to contribute to the development and maintenance of hypertension (Folkow, 1990; Pries et al., 1999).

The structural response to increased wall shear stress can also create positive feedback behavior. In a vessel with a fixed flow rate, this response generates negative feedback that regulates shear stress to a set point (Kamiya et al., 1984; Rodbard, 1975). However, the same response is destabilizing when two vessels are connected in parallel (Hacking et al., 1996). For example, if two vessels of the same length are fed by the same driving pressure, the vessel with the larger diameter experiences a larger wall shear stress and therefore tends to increase in diameter, while the smaller vessel shrinks and is eventually eliminated unless stabilized by metabolic stimuli (Pries et al., 1998). Furthermore, in a network with short and long flow pathways, the shear stress response tends to cause short channels to increase in diameter, forming functional shunts. Theoretical studies suggest that metabolic signals, acting in combination with upstream conducted responses, must be present to counteract this phenomenon (Pries et al., 1998, 2010).

The particulate nature of blood can also create positive feedback effects. In a diverging microvessel bifurcation, the daughter vessel with higher flow generally receives a higher hematocrit (Pries et al., 1989), and existing flow heterogeneity leads to a higher level of heterogeneity in erythrocyte fluxes. Oxygen saturation‐dependent ATP release from erythrocytes has been shown to act as a signaling mechanism for metabolic regulation of blood flow (Ellsworth et al., 2016). In a vessel branch receiving low flow, the hematocrit is reduced. Simulations suggest that this is destabilizing in the context of ATP signaling of hypoxia by erythrocytes, since vessels with low hematocrit and low oxygen levels are unable to generate ATP signals to increase flow (Fry & Secomb, 2021; Roy et al., 2012). Vessels with low or zero hematocrit can arise in heterogeneous networks under conditions of low demand, but as demand increases, the number of such vessels is predicted to decrease, representing a mechanism of capillary recruitment (Fry et al., 2013).

The presence of positive feedback loops in the control of blood flow tends to increase the heterogeneity of flow and tissue oxygenation. An appropriate balance between stabilizing negative and destabilizing positive feedback mechanisms is important for the control of heterogeneity.

### 3.5 Conducted versus local responses

In a network of vessels, the flow in any segment depends on all the diameters in the flow pathway involving that segment. In addition, the sensors and effectors involved lie at different points in the vascular network. Effective control of blood flow therefore requires coordinated changes in diameters of vessels along the flow pathway, implying the need for communication of control signals upstream and downstream (Secomb & Pries, 2002).

Acute flow regulation is achieved primarily by contraction and dilation of arterioles, since capillaries and venules have limited contractile ability. The upstream propagation of metabolic signals causing arteriolar vasodilation is a therefore critical component of local flow regulation (Segal, 2015). This communication is achieved by conducted responses along vessel walls. Locally generated metabolic signals in capillaries and terminal arterioles cause changes in endothelial cell membrane potential, which are transmitted upstream over millimeter distances via gap junctions connecting endothelial cells (Bearden et al., 2004; Gustafsson & Holstein‐Rathlou, 1999). Vascular smooth muscle cells also participate in conducted responses, and several signaling pathways are involved (Segal, 2015; Sinkler & Segal, 2017).

Long‐term structural adaptation of vessel diameters requires coordination of both upstream and downstream responses. In particular, such communication is essential to avoid the formation of functional shunts resulting from short flow pathways, as already discussed (Pries et al., 1998, 2010). Downstream communication can be accounted for by convective transport of metabolites and signal substances in the blood stream, while upstream communication depends on conducted responses.

In the lung, a mismatch of ventilation and perfusion, resulting from the heterogeneity of both variables, results in decreased oxygen uptake. Under conditions of high oxygen demand, such as exercise, hypoxic pulmonary vasoconstriction (HPV) acts to improve ventilation‐perfusion matching by reducing flow to poorly ventilated regions (Grimmer & Kuebler, 2017). Small pulmonary arterioles have sparse smooth muscle coverage and limited contractile ability; the results of simulations accounting for this imply an essential role for conducted responses in propagating signals to larger arterioles (Johnson et al., 2021).

Because conducted responses require continuous communication between endothelial cells over significant distances, they are vulnerable to disruption if endothelial cells are destabilized. Both acute and structural control mechanisms depend on conducted responses, and their ability to ameliorate the effects of heterogeneity is then compromised. This effect is hypothesized to underlie the structural disorganization that is typical of tumor vasculature (Pries et al., 2010). In the lung, disruption of endothelial communication has been observed during lung infection and endotoxemia, leading to impairment of the HPV response and ventilation‐perfusion matching, and to hypoxemia (Grimmer & Kuebler, 2017; Wang et al., 2012). In a study of blood flow control in skeletal muscle of young versus adult and elderly mice, microvascular architecture was preserved in aging, but impaired post‐contraction vasodilation was noted in older animals and attributed to impaired upstream conducted responses (Bearden et al., 2004).

## 4 DISCUSSION

Heterogeneity of structural and functional variables in the microcirculation is an inevitable consequence of the processes of microvascular growth and of the spatially and temporally varying demands placed upon it. This heterogeneity has generally detrimental effects on the performance of the system, particularly with regard to oxygen transport. Multiple control mechanisms of acute flow regulation and long‐term structural adaptation act to mitigate the effects of this heterogeneity, by improving the matching between oxygen delivery and demand in the systemic circulation and between ventilation and perfusion in the lungs.

Under normal resting conditions, the oxygen delivery system has substantial reserve capacity, and adequate tissue oxygenation is achieved without the need for tight control of flow distribution. However, under conditions of physiological stress, as for example in exercise, the need for close matching of supply with demand increases, and the role of local control mechanisms becomes more critical.

The presence of control mechanisms does not necessarily result in reduced heterogeneity in hemodynamic variables. A network with heterogeneous structure may require a high degree of heterogeneity in blood flow to achieve adequate tissue oxygenation. The mathematical analysis of heterogeneity of combined variables implies that control mechanisms may act to increase flow or capillary transit time heterogeneity while decreasing heterogeneity in functional parameters such as tissue oxygen levels.

These considerations lead to the conclusion that the functioning of local control mechanisms to mitigate the effects of heterogeneity is a basic requirement for normal tissue function, particularly under conditions of physiological stress. This conclusion has significant implications for the understanding and management of medical conditions in which tissue oxygenation is impaired. In the clinical setting, treatment approaches are often based on global parameter values without consideration of conditions at the microscopic level, where high levels of heterogeneity may exist. In pathophysiological conditions, as for example in sepsis, compromise of the regulatory mechanisms designed to compensate for heterogeneity can lead to organ dysfunction or failure (Bateman et al., 2015; Ellis et al., 2002; Roy & Secomb, 2021). Simply increasing cardiac output by increasing intravascular volume and stroke volume may not address issues of heterogeneity at the microvascular level. Similarly, increasing inspired oxygen concentration in the lung may do little to improve arterial oxygenation in acute respiratory distress syndrome if the severe ventilation‐perfusion mismatch is present.

In the heart, ischemia as manifested by angina can occur in spite of angiographically normal coronary arteries. This microvascular angina is thought to be associated with abnormal vasodilatory responses at the microcirculatory level as a consequence of endothelial dysfunction (Cannon & Epstein, 1988; Rahman et al., 2019).

In the brain, simulations have suggested that higher CBF may lead to decreased oxygen consumption due to shunting effects as flow increases, termed “malignant capillary transit time heterogeneity” (Angleys et al., 2015). Impairments in neurovascular coupling are present in various disease states such as stroke (Girouard & Iadecola, 2006), Alzheimer's disease (Kisler et al., 2017) and vascular dementia (Iadecola, 2013). In such cases, ischemic damage can be exacerbated by diversion of collateral flow away from injured areas (Jackman & Iadecola, 2015).

Systemic inflammatory states such as sepsis can cause disruption of endothelial function (Dolmatova et al., 2020), and in some cases undermine electrical coupling between endothelial cells mediated by connexins in gap junctions (Tyml, 2011). In such cases, reduction of inflammation or more specifically, restoration of inter‐endothelial coupling may serve as a therapeutic target to restore flow regulation and prevent hypoxia and organ damage.

The working range of acute flow regulation by arterioles is determined by the vessel wall properties. If vascular smooth muscle is fully relaxed, then further vasodilation is impossible, and the vessel is insensitive to additional vasodilator stimuli. A tissue in a vasodilated state has reduced capability to redistribute flow according to local needs. In this condition, a constitutive vasoconstriction may be beneficial, by restoring a regime in which regulatory mechanisms can respond to local tissue hypoxia. This, along with its ability to increase mean arterial pressure, provides an additional rationale for the use of vasopressors in critically ill patients.

However, vasopressor therapy can have deleterious effects on tissue perfusion by increasing afterload, which can result in decreased cardiac output and cardiac ischemia. Furthermore, therapeutic doses of vasopressors can cause decreased oxygen consumption and regional organ blood flow (Demiselle et al., 2020), particularly in the mesenteric and renal circulation (Hiltebrand et al., 2007; Malay et al., 2004). Different pressors may have varying abilities to restore systemic parameters while maintaining microcirculatory perfusion (van Loon et al., 2020).

In summary, extrinsic and intrinsic heterogeneities at the level of the microvasculature are inevitable. Both structural adaptation and flow regulation work in concert at different time scales to mitigate their effects and maintain oxygen uptake in the lung and delivery to tissue. This is accomplished by regulating blood flow to accommodate variations in oxygen demand as well as mitigating the effects of heterogeneity in the oxygen delivery system. Failure of these regulatory mechanisms can result in poor oxygen delivery even in the presence of adequate overall tissue perfusion. In pathophysiological conditions resulting in tissue hypoxemia and organ dysfunction, restoration of these regulatory mechanisms may be beneficial. This may take the form of restoring deficits in communication, for example by addressing inflammation and its effects on endothelial function, as well as restoring conditions under which these regulatory mechanisms are active by using pressors to modulate overall vascular tone. Increased understanding of the consequences of heterogeneity at the microvascular level may lead to therapeutic strategies to prevent organ failure in critically ill patients.
